# Aldehyde-functional thermoresponsive diblock copolymer worm gels exhibit strong mucoadhesion†

**DOI:** 10.1039/d2sc02074b

**Published:** 2022-05-26

**Authors:** Emma E. Brotherton, Thomas J. Neal, Daulet B. Kaldybekov, Mark J. Smallridge, Vitaliy V. Khutoryanskiy, Steven P. Armes

**Affiliations:** Dainton Building, Department of Chemistry, University of Sheffield Brook Hill Sheffield South Yorkshire S3 7HF UK s.p.armes@sheffield.ac.uk; School of Pharmacy, University of Reading, Whiteknights PO Box 224, Reading Berkshire RG6 6DX UK v.khutoryanskiy@reading.ac.uk; Department of Chemistry and Chemical Technology, Al-Farabi Kazakh National University Almaty 050040 Kazakhstan; GEO Specialty Chemicals, Hythe Southampton Hampshire SO45 3ZG UK

## Abstract

A series of thermoresponsive diblock copolymer worm gels is prepared *via* reversible addition–fragmentation chain transfer (RAFT) aqueous dispersion polymerization of 2-hydroxypropyl methacrylate using a water-soluble methacrylic precursor bearing pendent *cis*-diol groups. Selective oxidation using an aqueous solution of sodium periodate affords the corresponding aldehyde-functional worm gels. The aldehyde groups are located within the steric stabilizer chains and the aldehyde content can be adjusted by varying the periodate/*cis*-diol molar ratio. These aldehyde-functional worm gels are evaluated in terms of their mucoadhesion performance with the aid of a fluorescence microscopy-based assay. Using porcine urinary bladder mucosa as a model substrate, we demonstrate that these worm gels offer a comparable degree of mucoadhesion to that afforded by chitosan, which is widely regarded to be a ‘gold standard’ positive control in this context. The optimum degree of aldehyde functionality is approximately 30%: lower degrees of functionalization lead to weaker mucoadhesion, whereas higher values compromise the desirable thermoresponsive behavior of these worm gels.

## Abstract 2

Optimizing the aldehyde content of thermoresponsive diblock copolymer worm gels via periodate oxidation leads to mucoadhesion performance comparable to that of chitosan (a gold standard positive control) in a fluorescence assay using porcine mucosa.

## Graphical abstract

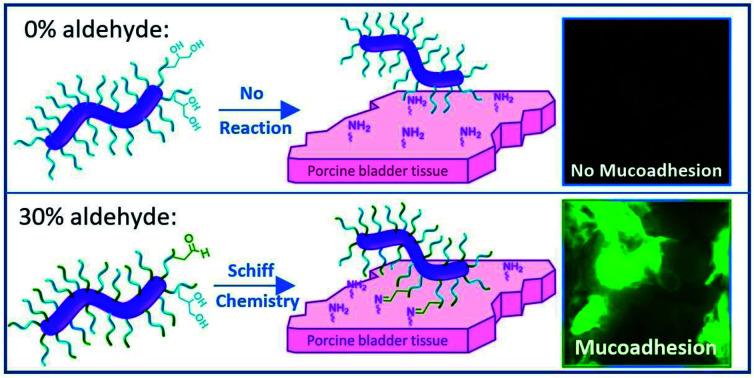

## Introduction

It is well known that amphiphilic diblock copolymers undergo spontaneous self-assembly in aqueous solution to form a wide range of nano-objects, including spheres, worms, vesicles or lamellae.^1–7^ Typically, such morphologies are accessed *via* post-polymerization processing *via* initial copolymer dissolution in a suitable water-miscible solvent such as THF or DMF, followed by dilution *via* slow addition of water.^8^ Under such near-equilibrium conditions, the precise copolymer morphology usually depends solely on the relative volume fraction of each block, as indicated by the fractional packing parameter originally introduced for conventional small molecule surfactants.^8–10^

Over the past decade or so, the development of polymerization-induced self-assembly (PISA) has provided convenient access to many copolymer morphologies.^11–22^ Unlike spheres or vesicles, diblock copolymer worms usually occupy relatively narrow phase space. Nevertheless, we and others have shown that the construction of pseudo-phase diagrams facilitates the reproducible synthesis of worms, which are usually well-defined in terms of their mean cross-sectional area but typically somewhat polydisperse in terms of their length.^12,18,20,23–31^

Such worms typically form 3D networks in semi-concentrated solution, which leads to macroscopic gelation under zero shear at ambient temperature.^25,26^ In particular, the reversible addition–fragmentation chain transfer (RAFT) aqueous dispersion polymerization of 2-hydroxypropyl methacrylate (HPMA) provides access to thermoresponsive worms that exhibit a worm-to-sphere transition on cooling to sub-ambient temperature.^27,32–35^ This morphological transition is accompanied by degelation and is reversible.^36^ This is important in the context of potential cell biology applications because it allows the media to be sterilized *via* ultrafiltration and enables the cells to be readily harvested after cell culture studies.^34,37–40^

Polymeric hydrogels have many applications in biomedical research, ranging from soft contact lenses to gel electrophoresis.^41,42^ In principle, hydrogels bearing appropriate chemical functionality can adhere to biological surfaces. This is likely to be particularly important for mucosal drug delivery, for which therapeutic efficiency is often substantially reduced by the continuous production and flow of biological fluids.^43,44^ This can result in drug leakage from the site of administration, which prevents effective localized delivery. For example, poor retention on mucosal surfaces is a common problem in delivering drugs to the eye, where the continuous production of tear fluid causes rapid removal of the active pharmaceutical ingredient from ocular surfaces.^45,46^ Similar problems are well-documented for the nasal cavity: the generation of mucus and the protective function afforded by mucociliary clearance does not allow drug molecules to be retained on the olfactory epithelium, which would otherwise potentially offers efficient nasal delivery to the brain.^47,48^ Similarly, drugs administered by catheter to treat bladder cancer also suffer from short residence times owing to the continuous production of urine and the periodic need for organ voiding.^49,50^

In principle, more effective drug delivery *via* mucosal surfaces should be feasible by designing mucoadhesive hydrogels. Various strategies to enhance mucoadhesion have been reported, including the design of copolymers containing thiol,^51,52^ acryloyl,^53,54^ methacryloyl^55,56^ or maleimide groups.^57,58^ These reactive moieties can form covalent bonds with the thiol group in cysteine, which is one of the amino acid building blocks present within mucins. Another strategy is the introduction of phenylboronic acid groups, which can form dynamic covalent bonds with the 1,2-diol-functional sugar groups expressed by mucins.^59,60^ Alternatively, catechol-based mucoadhesive polymers have been evaluated owing to their ability to form catechol-thiol or catechol-amine adducts with mucins.^61,62^ More recently, Bernkop-Schnürch and co-workers reported the synthesis of polymers functionalized with *N*-hydroxy(sulfo)succinimide esters that form amide bonds with mucins.^63,64^

Recently, we reported the use of RAFT polymerization^65–67^ for the synthesis of a new water-soluble methacrylic polymer (denoted as PGEO5MA) that contains pendent *cis*-diol groups, see Scheme S1.†^68^ This precursor can be oxidized under mild conditions in aqueous solution using sodium periodate to produce the corresponding aldehyde-functionalized water-soluble polymer. Subsequently, we employed a PGEO5MA precursor for the RAFT aqueous dispersion polymerization of HPMA to prepare a series of well-defined diblock copolymer spheres, worms or vesicles.^69^ In particular, it was shown that a model globular protein could be chemically adsorbed onto periodate-treated PGEO5MA-PHPMA vesicles *via* Schiff base chemistry (followed by *in situ* reduction of the initial labile imine linkages to produce hydrolytically stable secondary amine bonds). In the present study, we revisit this aqueous PISA formulation to prepare well-defined aldehyde-functional diblock copolymer worm gels and examine whether such materials offer any potential use in the context of mucoadhesion using porcine urinary bladder mucosa as a model system.

## Results and discussion

**Scheme 1:**
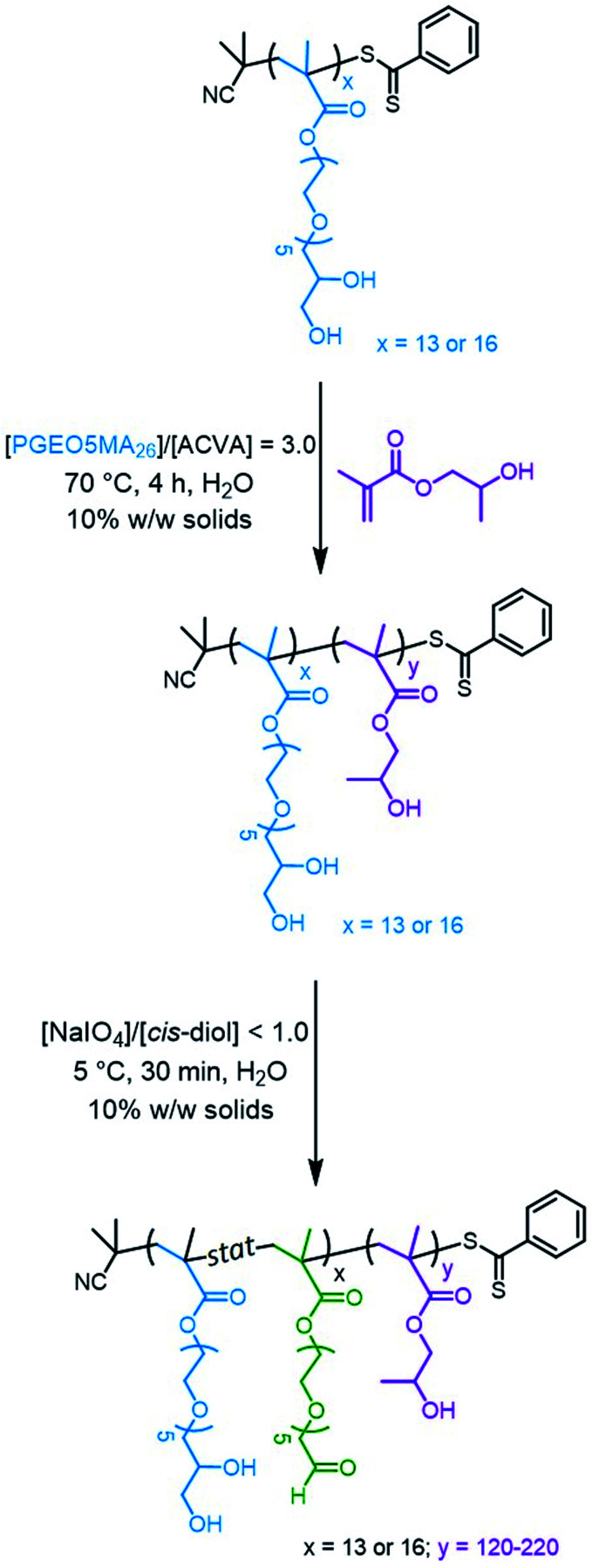
Two-step synthesis of aldehyde-functionalized PAGEO5MA_*x*_–PHPMA_*y*_ diblock copolymer worms starting from the *cis*-diol groups in the PGEO5MA_*x*_ homopolymer. In the first step, this water-soluble precursor is chain-extended *via* RAFT aqueous dispersion polymerization of HPMA. The second step involves partial selective oxidation of the PGEO5MA_*x*_ stabilizer block using aqueous sodium periodate at 22 °C.

Water-soluble PGEO5MA_13_ and PGEO5MA_16_ precursors were prepared *via* RAFT solution polymerization of GEO5MA in ethanol (Scheme S2†). DMF gel permeation chromatography (GPC) analysis (using a series of poly(methyl methacrylate) calibration standards) indicated that these homopolymers had *M*_n_ values of 9.3 and 11.2 kg mol^−1^, respectively, and relatively narrow molecular weight distributions (*Đ* = 1.19 and 1.18, respectively; Fig. S1†). Each PGEO5MA precursor was then chain-extended *via* RAFT aqueous dispersion polymerization of HPMA at 10% w/w solids (Scheme 1). A series of PGEO5MA_13_-PHPMA_*y*_ (*y* = 120–200) and PGEO5MA_16_-PHPMA_*y*_ (*y* = 140–220) diblock copolymer nanoparticles were prepared in order to identify a pure worm phase. All polymerizations had high HPMA conversions (>99%) as determined by ^1^H NMR spectroscopy while DMF GPC analysis indicated reasonably good RAFT control (*Đ* ≤ 1.25; Fig. S2†). A high molecular weight shoulder can be observed in each chromatogram, which has been previously attributed to dimethacrylate impurities in the HPMA monomer (<0.30 mol%).^70,71^ In particular, PGEO5MA_13_-PHPMA_150-190_ formed soft, thermoresponsive free-standing gels and a pure worm morphology was confirmed by transmission electron microscopy (TEM) studies (Fig. S3†). Similarly, a pure worm phase was obtained for PGEO5MA_16_-PHPMA_170–200_ as judged by TEM studies (Fig. S4†).

**Fig. 1:**
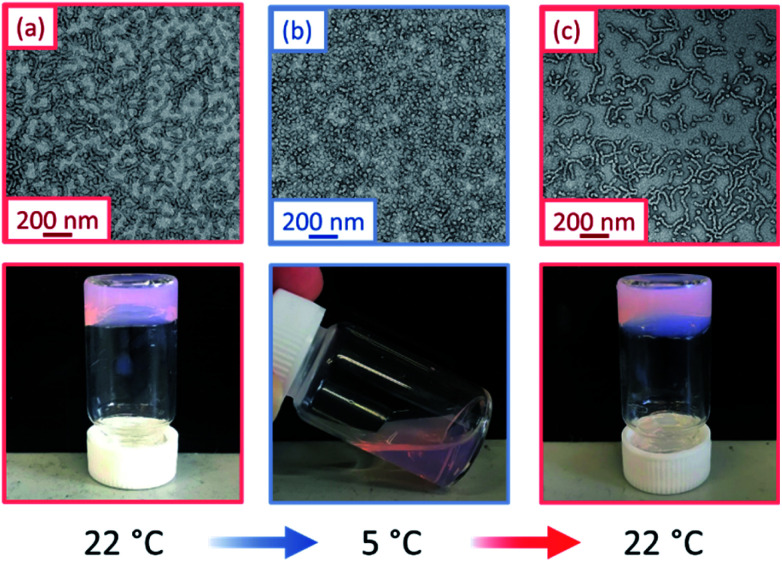
TEM images and corresponding digital photographs recorded for a 10% w/w aqueous dispersion of PGEO5MA_13_-PHPMA_155_ nano-objects: (a) soft, free-standing worm gel formed at 22 °C, (b) free-flowing fluid obtained on cooling to 5 °C and (c) the reconstituted worm gel formed after returning to 22 °C. [N.B. The pink coloration is conferred by the dithiobenzoate-based RAFT chain-ends].

The thermoresponsive nature of a PGEO5MA_13_-PHPMA_155_ and a PGEO5MA_16_-PHPMA_200_ worm gel was initially confirmed by visual inspection. A 10% w/w aqueous copolymer dispersion of each sample formed a soft, free-standing gel at 22 °C, see Fig. 1 and S5.† On cooling to 5 °C, degelation occurred to afford free-flowing liquids in both cases, with TEM analysis indicating a concomitant worm-to-sphere transition.

On returning to 22 °C, regelation was observed for PGEO5MA_13_-PHPMA_155_ owing to a sphere-to-worm transition. However, no regelation was observed for PGEO5MA_16_-PHPMA_200_, and TEM analysis indicated the presence of kinetically-trapped spheres and short worms in this case (Fig. S6†). Fully reversible thermoresponsive behavior is highly desirable for biomedical applications since this enables facile sterilization *via* cold ultrafiltration.^38^ Thus, only the PGEO5MA_13_-PHPMA_155_ worm gel was selected for the subsequent mucoadhesion studies.

**Fig. 2:**
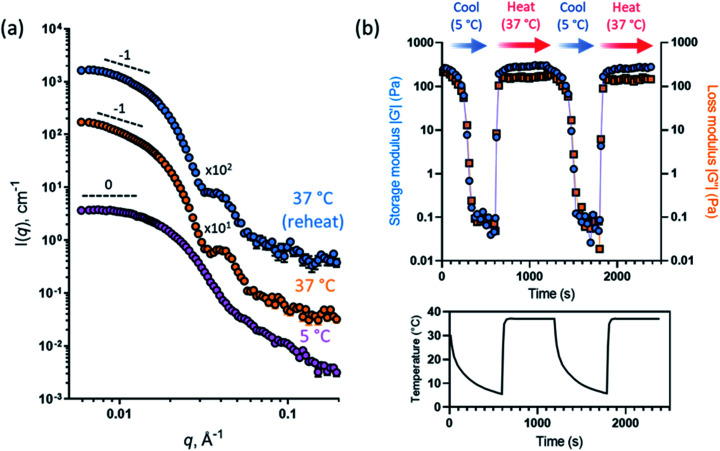
(a) SAXS patterns obtained for a 1.0% w/w aqueous dispersion of PGEO5MA_13_-PHPMA_155_ nano-objects initially at 37 °C (orange circles), after cooling to 5 °C (purple circles), and after returning to 37 °C (blue circles) [N.B. the two upper patterns are offset by the stated numerical factors to aid clarity]. Dashed lines indicate low *q* gradients of 0 and −1 as guidance for the eye, where such gradients indicate the presence of spheres and worms, respectively. (b) Storage and loss moduli (*G*′ and *G*′′, respectively) recorded for a 12% w/w aqueous dispersion of PGEO5MA_13_-PHPMA_155_ nano-objects over two 37 °C to 5 °C to 37 °C thermal cycles using oscillatory rheology. A temperature–time profile for such experiments is also displayed.

This thermally-induced morphological transition was further characterized using small-angle X-ray scattering (SAXS). A 1.0% w/w aqueous dispersion of PGEO5MA_13_-PHPMA_155_ worms was studied at 37 °C and 5 °C. At 37 °C, a gradient of −1 was observed in the Guinier region (low *q*) of the SAXS pattern (Fig. 2a), which is consistent with the highly anisotropic worms observed by TEM (Fig. 1). However, a gradient of zero is observed in the same low *q* region on cooling to 5 °C (Fig. 2a). This indicates the presence of spherical nanoparticles, which agrees with the TEM image recorded at the same temperature (Fig. 1). Finally, the 1.0% w/w dispersion was warmed to 37 °C and allowed to equilibrate at this temperature for 1 h. The SAXS pattern recorded after equilibration is almost identical to that original pattern acquired at 37 °C (Fig. 2a). This demonstrates that these PGEO5MA_13_-PHPMA_155_ nano-objects exhibit thermoreversible behavior with minimal hysteresis. Moreover, the core radii for the worms (*r*_w_) and the spheres (*r*_s_) can be estimated using *r*_w_ = 3.83/*q* and *r*_s_ = 4.49/*q* respectively, where *q* corresponds to the intensity minimum. The approximate core radii for the initial worm gel, the cold spheres, and the reconstituted worm gel are calculated to be 11, 10, and 12 nm, respectively. These values are comparable with core radii estimated by TEM analysis (counting at least 100 nanoparticles per sample).

The PGEO5MA_13_-PHPMA_155_ worm gel was lyophilized to produce a freeze-dried powder. Redispersion of this copolymer powder in ice-cold deionized water (which ensures near-molecular dissolution of the amphiphilic copolymer chains^72^) followed by warming to 22 °C produced a soft, free-standing worm gel. Empirically, it was found that redispersion at 12% w/w solids produced longer, more linear worms than redispersion at 10% w/w solids. Thus, all subsequent experiments were conducted at 12% w/w solids.

Oscillatory rheology was used to characterize the thermoresponsive behavior of a 12% w/w aqueous dispersion of PGEO5MA_13_-PHPMA_155_ nano-objects. This sample was subjected to shear (1.0% strain at an angular frequency of 1 rad s^−1^; Fig. 2b) over two thermal cycles (from 5 °C to 37 °C to 5 °C). On cooling to 5 °C the initial worms are converted into spheres, which causes *in situ* degelation (*G*′′ > *G*′; Fig. 2b) and a significant reduction in the complex viscosity (from 345 Pa s at 37 °C to 0.09 Pa s at 5 °C). On warming to 37 °C, worms are reformed and regelation occurs (*G*′′ < *G*′; Fig. 2b), with the complex viscosity increasing to 347 Pa s. Essentially the same (de)gelation behavior was observed during the second thermal cycle, which indicates excellent thermoreversibility.

Importantly, the pendent *cis*-diol units on these copolymer worms can be selectively oxidized using sodium periodate in aqueous solution under mild conditions to introduce aldehyde groups within the steric stabilizer chains (Scheme 1).^68,69^ In principle, such derivatization might be expected to produce mucoadhesive worm gels since it is well-known that aldehydes can react readily with amines *via* Schiff base chemistry.^73^ However, fluorescence labelling is normally required for mucoadhesion flow-through assays.^74,75^ Therefore, HPMA and fluorescein methacrylate (FMA) were statistically copolymerized to produce fluorescein-tagged worms (Scheme S3†). A FMA content of 0.1 mol% was targeted and the overall comonomer conversion was more than 99% as indicated by ^1^H NMR spectroscopy. The initial pink worm gel formed a bright yellow worm gel on adjusting the solution from pH 5 to pH 9 with NaOH, indicating that the FMA was incorporated within the core-forming block (Fig. S7†). Moreover, UV GPC analysis performed at *λ* = 495 nm (which corresponds to the maximum absorbance for the FMA repeat units when they are in their anionic carboxylate form) produced a very similar molecular weight distribution curve to that recorded using a refractive index detector (Fig. S8†).^76^ TEM analysis confirmed that there was no discernible difference between the fluorescently-labeled PGEO5MA_13_-P(HPMA_155_-*stat*-FMA_0.15_) worms and the non-fluorescent PGEO5MA_13_-PHPMA_155_ worms (Fig. S9†).

The former worms were subsequently oxidized with sodium periodate targeting periodate/*cis*-diol molar ratios of 0.10, 0.20, 0.30 or 0.50 using a previously reported protocol developed for crosslinked PGEO5MA_26_-PHPMA_350_-PEGDMA_20_ vesicles.^69^ In each case, the extent of oxidation was determined by ^1^H NMR spectroscopy (Fig. S10†). We chose to conduct periodate oxidation on the final diblock copolymer nanoparticles rather than on the PGEO5MA precursor. This approach means that the same *cis*-diol-functional worm gel precursor was used to produce each aldehyde-functional worm gel examined in this study, which eliminates batch-to-batch variability. Moreover, ^1^H NMR spectroscopy studies (data not shown) confirmed that a sample of 100% aldehyde-functional PAGEO5MA_26_-PHPMA_250_ worms reported in our prior study^69^ remained stable with respect to aerial oxidation for at least one year when stored at ambient temperature. TEM studies confirmed that there was no discernible change in morphology after oxidation (Fig. S9 and S11†). Furthermore, oscillatory strain sweeps (from 0.1% to 20%) were performed on these partially oxidized worm gels to assess how the degree of aldehyde functionality affected the gel properties (Fig. S12†). It was found that a higher degree of oxidation led to a lower strain being required for degelation (*G*′′ < *G*′). For example, degelation of the *cis*-diol functional precursor worm gel required an applied strain of 16.8%, whereas the 50% aldehyde-functionalized worm gel underwent degelation at just 1.1% strain (Fig. S12†). Moreover, higher degrees of aldehyde functionalization led to higher gel viscosities (Fig. S13†). For example, the gel viscosity of the *cis*-diol functional precursor worm gel was 72 Pa s at an applied strain of 1.0% when equilibrated at ambient temperature, whereas the 50% aldehyde-functionalized worm gel exhibited a gel viscosity of 263 Pa s under the same conditions (Fig. S13†). This indicates that the introduction of aldehyde groups produces stronger (but more fragile/brittle) gels. There are multiple two possible for why the gels become stronger with increased aldehyde content. In principle, the pendent aldehyde groups can react with the remaining *cis*-diols to form hemiacetal bonds between neighboring worms, thus leading to stronger gels. However, GPC analysis (see Fig. S14†) of the aldehyde-functional diblock copolymer chains provides no evidence for inter-chain crosslinking, which would produce a high molecular weight shoulder. Alternatively, the higher storage moduli observed for the aldehyde-functional worm gels may be related to the formation of stronger hydrogen bonds between the hydroxyl (or ester carbonyl) groups on the remaining *cis*-diol repeat units and the aldehyde groups (or geminal diol) groups. Further studies (perhaps with model compounds) are required to answer this question but this is beyond the scope of the present study.

**Fig. 3:**
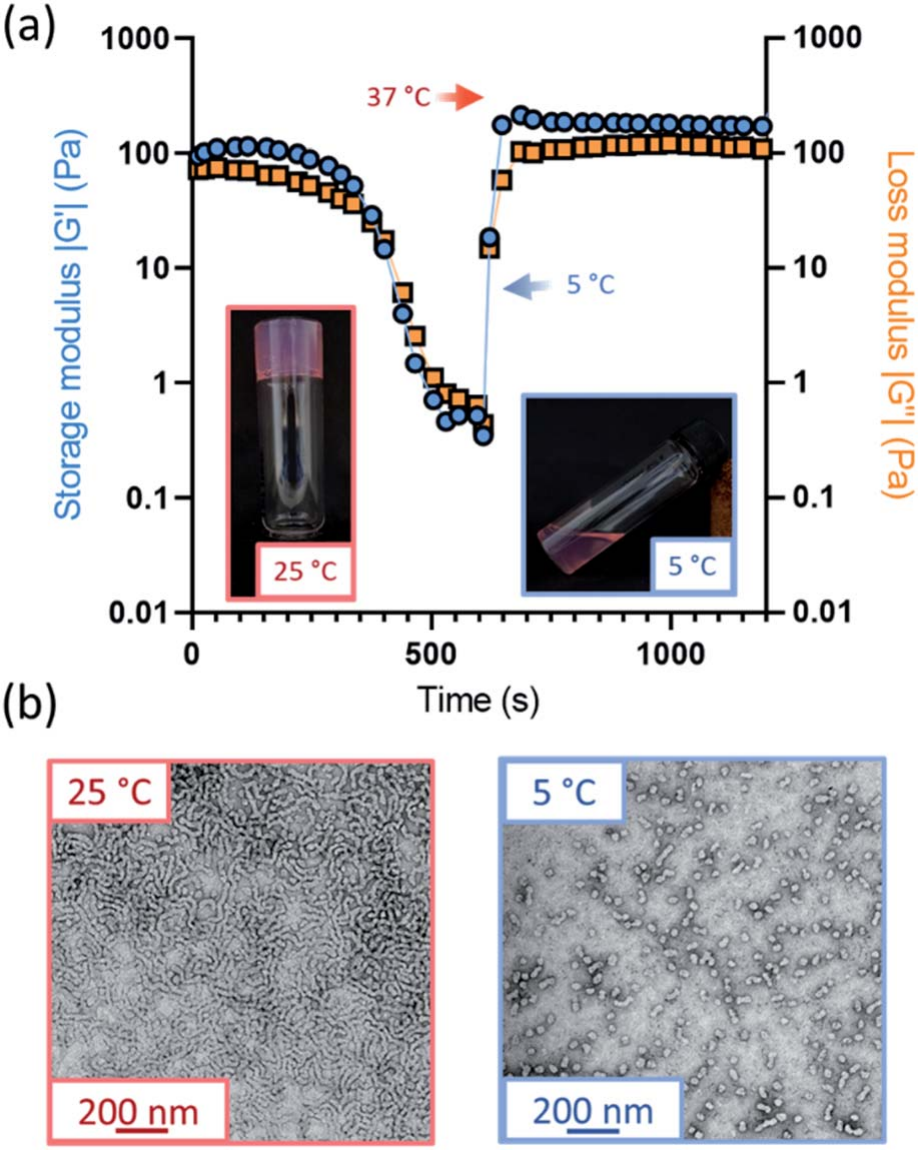
(a) Variable temperature oscillatory rheology monitoring the storage and loss modulus (*G*′ and *G*′′, respectively) at both 5 °C and 37 °C for PGEO5MA_13_-P(HPMA_155_-*stat*-FMA_0.15_) worms with 30% aldehyde functionality. (b) TEM images for PGEO5MA_13_-P(HPMA_155_-*stat*-FMA_0.15_) worms with 30% aldehyde at 25 °C and the spherical nanoparticles at 5 °C.

Further variable temperature oscillatory rheology experiments were performed on the oxidized worm gels, whereby samples were first cooled to 5 °C and subsequently heated to 37 °C under an applied shear (Fig. 3a and S15†). Thermoreversible degelation was observed in most cases but the rate of degelation was notably slower for gels with higher degrees of aldehyde functionality. Such thermoresponsive behavior was confirmed by visual inspection: free-standing gels became free-flowing liquids after cooling from 22 °C to 5 °C for 50 min and reformed free-standing gels on returning to ambient temperature (Fig. S16†). These observations were consistent with TEM studies, which indicated the presence of spheres at 5 °C and worms at 25 °C (Fig. 3b). However, the 50% aldehyde-functional worm gel did not undergo degelation at all on the timescale (20 min) of the rheology experiments (Fig. S15d†). Thus, the highest degree of aldehyde functionality that can be incorporated into the PGEO5MA_13_-P(HPMA_155_-*stat*-FMA_0.15_) worm gel precursor without significantly affecting its thermoresponsive behavior is 30%.Interestingly, the worm gels remained pink after oxidation, suggesting retention of the dithiobenzoate end-groups (Fig. S16†). This was confirmed by UV GPC studies (*λ* = 298 nm), which indicated that this RAFT end-group is retained after periodate oxidation (Fig. S14†).

**Fig. 4:**
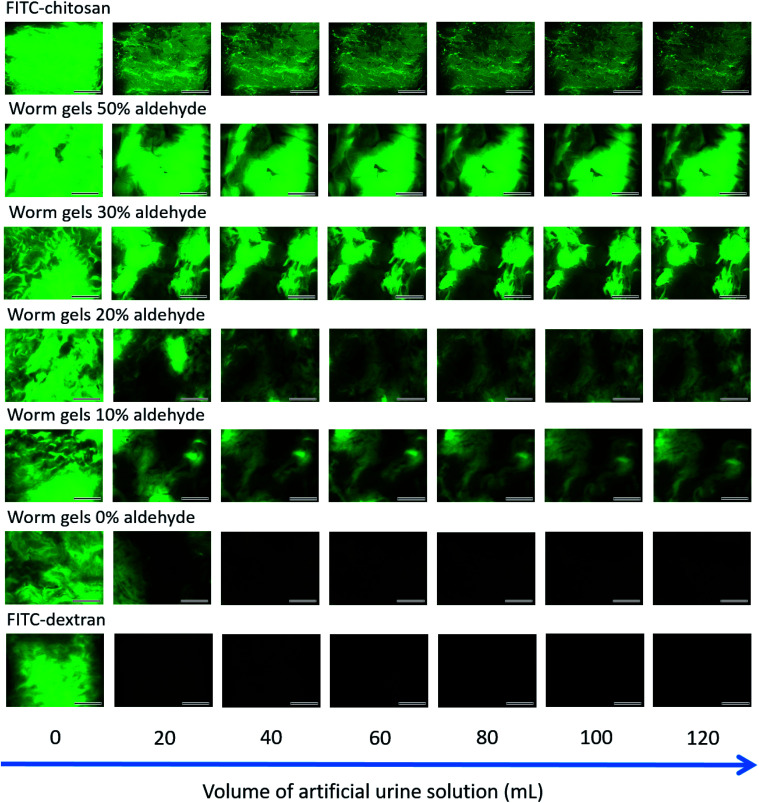
Representative fluorescence images of freshly-dissected porcine urinary bladder mucosa illustrating the retention of fluorescently-labeled PGEO5MA_13_-P(HPMA_155_-*stat*-FMA_0.15_) worm gels bearing varying degrees of aldehyde functionality after irrigation with varying volumes of AU solution at a flow rate of 2.0 mL min^−1^, plus positive and negative controls (FITC-chitosan and FITC-dextran, respectively). Scale bars correspond to 6 mm.

**Fig. 5:**
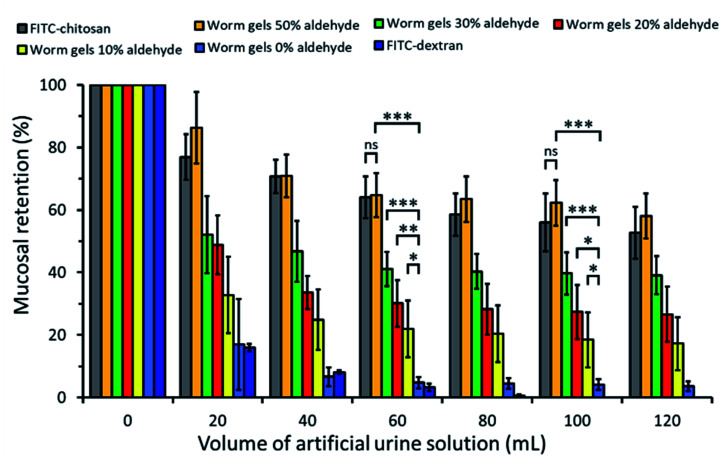
Percentage retention for fluorescently-labeled PGEO5MA_13_-P(HPMA_155_-*stat*-FMA_0.15_) worm gels with differing degrees of aldehyde functionality on freshly dissected porcine urinary bladder mucosa after irrigating with varying volumes of AU solution at a flow rate of 2.0 mL min^−1^, plus positive and negative controls. Data are expressed as mean values ± standard deviations (*n* = 3). Statistically significant differences are given as: * = *p* < 0.05; ** = *p* < 0.01; *** = *p* < 0.001; ns denotes no significance.

The retention of such worm gels on mucosal surfaces was studied using a porcine urinary bladder mucosa model under a constant flow of artificial urine (AU). This model mimics the physiologically relevant conditions within the urinary bladder following the intravesical administration of therapeutic agents for the treatment of bladder cancer or interstitial cystitis. Fig. 4 shows fluorescence images recorded for urinary bladder tissue when using a series of fluorescently-labeled worm gels plus two control samples after washing with varying volumes of AU. Fluorescein isothiocyanate (FITC)-chitosan and FITC-dextran were used as positive and negative controls owing to their strong and weak adhesion to mucosal tissues, respectively.^77^ Worm gels bearing 0, 10, 20, 30 or 50 mol% aldehyde functionality were evaluated in these experiments. Visual inspection of these images indicates that the incorporation of aldehyde groups within such worm gels clearly improves their retention on mucosal tissue. All images were analyzed using ImageJ software to determine fluorescence intensities, which were then converted into % mucosal retention (Fig. 5). This approach enables quantitative interpretation of the wash-off experiments. The worm gel containing no aldehyde groups exhibited relatively weak adhesion to the mucosa, which is initially comparable to that for FITC-dextran. However, unlike this negative control, this worm gel are still retained on mucosal surface to some extent even after washing with up to 120 mL of AU. This may be related to their favorable rheological characteristics relative to non-gelling FITC-dextran. The worm gel containing 10% aldehyde groups exhibited substantially improved retention with around 20% remaining on the bladder mucosa after washing with 120 mL of AU. Further increasing the aldehyde content in the worm gels up to either 20 or 30% led to progressively stronger mucoadhesion. Most notably, the worm gel bearing 50% aldehyde groups exhibited comparable mucoadhesion to that of chitosan, which is widely considered to be a ‘gold standard’ mucoadhesive polymer.^78^ It is perhaps worth emphasizing that such polyelectrolytes usually exhibit superior mucoadhesive properties compared to non-ionic polymers.^78^ In contrast, the hydroxyl-rich worm gels examined in this study possess solely non-ionic character.

## Experimental

### Materials

GEO5MA monomer was synthesized at GEO Specialty Chemicals (Hythe, UK) by Dr C. P. Jesson as previously reported.^68^ 2-Hydroxypropyl methacrylate (HPMA, 97%) was provided by GEO Specialty Chemicals (Hythe, UK). 4,4′-Azobis(4-cyanopentanoic acid) (ACVA; >98%), sodium periodate (NaIO_4_, ≥99.8%), chitosan (low molecular weight), fluorescein methacrylate (FMA; 95%), fluorescein isothiocyanate (FITC) (isomer l) and FITC-dextran (MW = 3000–5000 Da) were purchased from Sigma-Aldrich (UK). 2-Cyano-2-propyl dithiobenzoate (CPDB, >97%) was purchased from Strem Chemicals Ltd (Cambridge, UK). Ethanol and diethyl ether were purchased from Fisher Scientific (UK). *d*_7_-Dimethylformamide (DMF) was purchased from Goss Scientific Instruments Ltd (Cheshire, UK). All reagents were used as received unless otherwise stated. Dialysis tubing with a molecular weight cut-off of 12 000–14 000 Da was purchased from Medicell Membranes Ltd. (UK). Deionized water was used for all experiments involving aqueous solutions.

### Methods

#### ^1^H NMR spectroscopy

Spectra were recorded in *d*_7_-DMF using a 400 MHz Bruker Avance-400 spectrometer at 298 K with 16 scans being averaged per spectrum.

#### DMF gel permeation chromatography (GPC)

DMF GPC was used to determine the number-average molecular weight (*M*_n_) and dispersity (*Đ*) for all homopolymers and diblock copolymers. The instrument set-up comprised two Agilent PL gel 5 μm Mixed-C columns and a guard column connected in series to an Agilent 1260 Infinity GPC system operating at 60 °C. The GPC eluent was HPLC-grade DMF containing 10 mmol LiBr at a flow rate of 1.0 mL min^−1^, the copolymer concentration was typically 1.0% w/w, and calibration was achieved using a series of ten near-monodisperse poly(methyl methacrylate) standards ranging from 1080 g mol^−1^ to 905 000 g mol^−1^. Chromatograms were analyzed using Agilent GPC/SEC software.

#### Rheology

An AR-G2 rheometer equipped with a variable temperature Peltier plate and a 40 mm 2° aluminum cone was used for all rheological experiments. Preliminary strain sweep experiments were performed on worm gels at 0.1% to 20% strain and a constant angular frequency of 1.0 rad s^−1^ to assess their gel strength and to identify the linear viscoelastic region. Subsequently, the storage modulus (*G*′), loss modulus (*G*′′) and complex viscosity (|η*|) were determined as a function of temperature at an applied strain of 1.0% and an angular frequency of 1.0 rad s^−1^. The gels were initially cooled to 5 °C for 10 min, prior to heating to 37 °C and allowing 10 min for thermal equilibrium at the latter temperature. Rheology measurements were performed during this thermal cycle at 1.0% strain and an angular frequency of 1.0 rad s^−1^.

#### Transmission electron microscopy (TEM)

Copper/palladium TEM grids (Agar Scientific, UK) were coated in-house to yield a thin film of amorphous carbon. The grids were subjected to a glow discharge for 30 s. For each sample, a 5.0 μL droplet of a 0.1% w/w aqueous copolymer dispersion was placed on a freshly-treated grid for 1 min and carefully blotted with filter paper to remove excess solution. Then a 5.0 μL droplet of a 0.75% w/w aqueous uranyl formate solution was placed on the sample-loaded grid for 20 s and blotted with filter paper to remove excess stain. This negative staining protocol was required to ensure sufficient electron contrast. Each grid was then dried using a vacuum hose. Imaging was performed at 80 kV using an FEI Tecnai Spirit 2 microscope fitted with an Orius SC1000B camera.

#### Small-angle X-ray scattering (SAXS)

SAXS patterns were recorded using a Xeuss 2.0 laboratory beamline (Xenocs, Grenoble, France) equipped with a 2D Pilatus 1 M pixel detector (Dectris, Baden-Daettwil, Switzerland) and a MetalJet X-ray source (Ga Kα radiation, *λ* = 1.34 Å; Excillum, Kista, Sweden). The scattering vector range was 0.006 Å^−1^ < *q* < 0.2 Å^−1^, where 
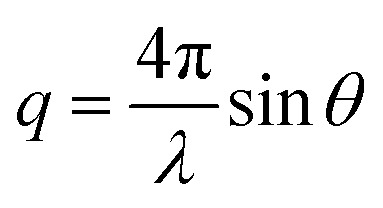
 and *θ* is half of the scattering angle. Glass capillaries of 2.0 mm diameter were used as a sample holder and the sample temperature was controlled using a HFSX350-CAP heating/cooling stage (Linkam Scientific Instruments Ltd, Tadworth, UK), with 10 min being allowed for thermal equilibration of each capillary prior to data collection. 2D X-ray scattering patterns were reduced using software supplied by the SAXS instrument manufacturer. Background subtraction and further data analysis were performed using Irena SAS macro (version 2.61) for Igor Pro.^79^ The scattering of pure water was used for absolute intensity calibration of the SAXS patterns.

### *Ex vivo* mucoadhesion studies on porcine urinary bladder tissues

#### Flow-through technique

Porcine urinary bladder tissues were received from P.C. Turner Abattoirs (Farnborough, UK) immediately after animal slaughter and used within 24 h. Such bladder tissue was used to evaluate mucosal retention of the worm gels (or chitosan) using an established flow-through method involving fluorescence detection.^74,77,80^ Tissues were carefully dissected (avoiding contact with the internal mucosa) using disposable sharp blades to yield 2 × 2 cm sections, which were then used for the experiments. Dissected bladder tissue was mounted on a glass slide with the mucosal side facing upward and pre-rinsed with 3.0 mL of artificial urine (AU) solution (pH 6.20) before commencing each *ex vivo* mucoadhesion test. Experiments to assess the retention of each worm gel on urinary bladder mucosa were performed at 37 °C and 100% relative humidity within an incubator. Fluorescence images were recorded for the mucosal surface of the bladder using a Leica MZ10F stereomicroscope (Leica Microsystems, UK) equipped with a Leica DFC3000G digital camera fitted with a green fluorescence protein filter at 1.25× magnification using an exposure time of 485 ms and a 2.0× gain. Initially, images of bare bladder tissue (without any test material) were acquired to determine the background fluorescence intensity for each sample.

Aqueous solutions of FITC-chitosan (1.0 mg mL^−1^ in 0.5% acetic acid) and FITC-dextran (1.0 mg mL^−1^ in deionized water) were prepared for use as positive and negative controls, respectively. The FITC-chitosan solution was adjusted to pH 6.0 using 0.1 M NaOH. Then a 200 μL aliquot of either a fluorescently-labeled 12% w/w PGEO5MA_13_-P(HPMA_155_-*stat*-FMA_0.15_) worm gel prepared in deionized water or a control sample was pipetted onto a mucosal surface and repeatedly washed with AU solution at a flow rate of 2.0 mL min^−1^ using a syringe pump (total washing time was 60 min). A microscopy image of the mucosal surface of each bladder sample was collected at predetermined time points and then analyzed with ImageJ software by measuring the pixel intensity after each wash. The pixel intensity of the control samples was subtracted from each measurement to obtain normalized intensities. Images from control samples were collected using an exposure time of 20 ms at 1.0× gain. All measurements were conducted in triplicate.

#### Statistical analysis

Mucoadhesion data (expressed as mean values ± standard deviations) were calculated and assessed for significance using a two-tailed Student's *t*-test and a one-way analysis of variance followed by the Bonferroni post hoc test using GraphPad Prism software (version 7.0), where *p* < 0.05 was taken to be significant.

### Synthesis

#### Synthesis of PGEO5MA_*x*_ precursors by RAFT solution polymerization in ethanol

A PGEO5MA_13_ and a PGEO5MA_16_ precursor were prepared in this study. The synthesis of PGEO5MA_13_ is representative of the general protocol. GEO5MA monomer (25.0 g, 65.7 mmol), CPDB RAFT agent (1.45 g, 6.57 mmol), ACVA initiator (0.368 g, 1.31 mmol; CTA/initiator molar ratio = 5.0) and ethanol (17.9 g) were weighed into a 100 mL round-bottom flask. The reaction mixture was deoxygenated for 40 min using a stream of N_2_ gas before immersing the flask in an oil bath set at 70 °C for 180 min. The polymerization was quenched by removing the flask from the oil bath and cooling to 20 °C while simultaneously exposing the reaction mixture to air. The GEO5MA conversion was determined to be 85% by ^1^H NMR spectroscopy (the residual monomer vinyl signals at 5.61–6.18 ppm were compared to the five methacrylic backbone protons at 0.78–2.71 ppm). The crude precursor was purified by precipitation into excess diethyl ether to remove any unreacted monomer and other impurities, followed by filtration and redissolution in methanol. This precipitation step was repeated and the purified homopolymer was dried in a vacuum oven set at 35 °C overnight to produce a red viscous liquid. The mean DP of this precursor was determined to be 13 by end-group analysis using ^1^H NMR spectroscopy (the five aromatic protons of the dithiobenzoate chain-end at 7.34–8.03 ppm were compared to the five methacrylic backbone protons at 0.78–2.71 ppm.

#### Synthesis of PGEO5MA_*x*_-PHPMA_*y*_ diblock copolymer nanoparticles by RAFT aqueous dispersion polymerization of HPMA

PGEO5MA_13_-PHPMA_*y*_ and PGEO5MA_16_-PHPMA_*y*_ nanoparticles were prepared at 10% w/w solids. The synthesis of PGEO5MA_13_-PHPMA_150_ is representative of the general protocol. HPMA monomer (0.500 g, 3.47 mmol), PGEO5MA_13_ precursor (120 mg, 23.1 μmol; target PHPMA DP = 150), ACVA initiator (2.20 mg, 7.71 μmol; PGEO5MA_13_/initiator molar ratio = 3.0) and water (5.59 g) were weighed into a 15 mL sample vial. The reaction mixture was deoxygenated using a stream of N_2_ gas for 30 min and the sample vial was placed into an oil bath set at 70 °C. After 4 h, the vial was removed from the oil bath and the polymerization was quenched by cooling to 20 °C while exposing the contents of the vial to air. The final HPMA conversion was determined to be 99% by ^1^H NMR spectroscopy (the residual monomer vinyl signals at 5.61–6.18 ppm were compared to the integrated methacrylic backbone signals at 0.81–2.30 ppm).

#### Synthesis of PGEO5MA_13_-P(HPMA_155_-*stat*-FMA_0.15_) diblock copolymer nanoparticles by RAFT aqueous dispersion copolymerization of HPMA with FMA

Fluorescently-labeled PGEO5MA_13_-P(HPMA_155_-*stat*-FMA_0.15_) nanoparticles were prepared at 10% w/w solids. HPMA monomer (3.00 g, 20.8 mmol), FMA (8.30 mg, 20.8 μmol) and PGEO5MA_13_ precursor (0.694 g, 134 μmol; target PHPMA DP = 155) were added in turn to a 100 mL round-bottom flask and stirred until a homogeneous solution was obtained. Then ACVA initiator (7.50 mg, 26.9 μmol; PGEO5MA_13_/ACVA molar ratio = 5.0) and water (27.2 g) were added to the flask and the reaction mixture was deoxygenated using a stream of N_2_ gas for 30 min prior to immersing the flask in an oil bath set at 70 °C. After 4 h, the copolymerization was quenched by cooling the flask to 20 °C while simultaneously exposing the contents of the flask to air. The final HPMA conversion was determined to be 99% by ^1^H NMR spectroscopy (the integrated monomer vinyl signals at 5.67–6.16 ppm were compared to the methacrylic backbone protons at 0.81–2.30 ppm). Copolymers were dialyzed against methanol for 24 h and then deionized water for two days.

#### Selective oxidation of PGEO5MA_13_-P(HPMA_155_-*stat*-FMA_0.15_) diblock copolymer nanoparticles using **sodium periodate**

The synthesis of PGEO5MA_13_-P(HPMA_155_-*stat*-FMA_0.15_) nanoparticles with 10% aldehyde functionality in aqueous solution is representative of the general protocol. Sodium periodate (1.50 mg, 7.14 μmol) was dissolved in a 12% w/w aqueous dispersion of PGEO5MA_13_-P(HPMA_155_-*stat*-FMA_0.15_) nanoparticles (3.00 g, 0.11 mmol) that had been pre-cooled to 5 °C. A periodate/*cis*-diol molar ratio of 0.10 was used to target a degree of aldehyde functionality of 10%. The periodate oxidation reaction was conducted in the dark at 5 °C for 30 min with continuous stirring [N.B. Under such conditions, the PGEO5MA_13_-P(HPMA_155_-*stat*-FMA_0.15_) chains form spherical nanoparticles as opposed to worms, which is beneficial for efficient stirring]. The degree of aldehyde functionality was determined to be approximately 10% by ^1^H NMR spectroscopy (the geminal diol signal assigned to the AGEO5MA units at 5.13 ppm was compared to the five methacrylic backbone protons at 0.81–2.30 ppm). Other degrees of aldehyde functionality were targeted by adjusting the periodate/*cis*-diol molar ratio as required. Periodate-treated copolymers were dialyzed against deionized water for two days.

#### Synthesis of FITC-labeled chitosan

Chitosan was labeled with FITC using a previously reported protocol.^81,82^ First, chitosan (1.00 g) was dissolved in 0.10 M acetic acid (100 mL), stirred overnight and vacuum-filtered to remove any undissolved chitin particles. Then FITC (100 mg) dissolved in methanol (50 mL) was added to the remaining aqueous acidic solution of chitosan and the resulting reaction mixture was stirred in the dark at 20 °C for 3 h. The FITC-labeled chitosan was then precipitated into 0.10 M NaOH. The insoluble product was isolated by filtration, redissolved in water and purified by dialysis against deionized water (5 L; nine changes) in the dark to remove any unreacted FITC. Finally, the dialyzed product was lyophilized overnight. The resulting FITC-chitosan was placed in an amber vial wrapped with aluminum foil to exclude light and stored in a refrigerator prior to use.

## Conclusions

We report the synthesis of thermoresponsive diblock copolymer worm gels *via* RAFT aqueous dispersion polymerization of HPMA using a water-soluble methacrylic precursor bearing pendent *cis*-diol groups. Selective oxidation using an aqueous solution of sodium periodate introduces aldehyde groups within the steric stabilizer chains and the aldehyde content can be readily adjusted by varying the NaIO_4_/*cis*-diol molar ratio. A series of such aldehyde-functional worm gels are evaluated in the context of mucoadhesion using porcine urinary bladder as a model substrate. A bespoke fluorescence microscopy assay demonstrates that such worm gels can offer similar performance as that afforded by chitosan, which is widely employed as a ‘gold standard’ positive control in this field. One potentially important advantage of these worm gels over chitosan is their non-ionic character, which should enable potential compatibility problems (*e.g.*, complexation with anionic drugs) to be avoided. The optimum degree of aldehyde functionality is approximately 30%: lower degrees of functionalization lead to significantly weaker mucoadhesion, whereas higher values compromise the desirable thermoresponsive behavior of these worm gels. In summary, aldehyde-functionalized worm gels represent a new family of strongly mucoadhesive polymers that can form dynamic covalent imine bonds with mucosal membranes under physiological conditions.

## Data availability

Relevant additional data are provided in the ESI.†

## Author contributions

E. E. B. and T. J. N. prepared all the diblock copolymer worms and performed NMR and GPC analyses. E. E. B conducted the periodate oxidation experiments and analysed the nanoparticles by TEM. T. J. N. characterized the worms by SAXS and performed the gel rheology experiments. D. B. K. performed all the mucoadhesion studies. S. P. A. and V. V. K. conceived the project, M. J. S. provided partial funding and all authors contributed to writing this manuscript.

## Conflicts of interest

There are no conflicts to declare.

## Supplementary Material

SC-013-D2SC02074B-s001
