# Supplementary material for: Aldehyde-functional thermoresponsive diblock copolymer worm gels exhibit strong mucoadhesion
Source: Chem Sci. 2022 May 26;13(23):6888–98. doi: 10.1039/d2sc02074b (PMC9200053; doi:10.1039/d2sc02074b)
Supplement: SC-013-D2SC02074B-s001 [file SC-013-D2SC02074B-s001.pdf]

Electronic Supplementary Material (ESI) for Chemical Science.
This journal is © The Royal Society of Chemistry 2022

Supporting Information for:

Aldehyde-functional Thermoresponsive Diblock Copolymer Worm Gels Exhibit Strong Mucoadhesion

Emma E. Brotherton,[§,a] Thomas J. Neal,[§,a] Daulet B. Kaldybekov,[§,b,c]

Mark J. Smallridge,[d] Vitaliy V. Khutoryanskiy[b,*] and Steven P. Armes[a,*]

a. Dainton Building, Department of Chemistry, University of Sheffield,

Brook Hill, Sheffield, South Yorkshire, S3 7HF, UK.

b. School of Pharmacy, University of Reading, Whiteknights,

PO Box 224, Reading, Berkshire, RG6 6DX, UK.

c. Department of Chemistry and Chemical Technology, Al-Farabi Kazakh National University

Almaty 050040, Kazakhstan

d. GEO Specialty Chemicals, Hythe, Southampton, Hampshire SO45 3ZG, UK.

* To whom correspondence should be addressed (s.p.armes@shef.ac.uk and v.khutoryanskiy@reading.ac.uk)

[§] These authors contributed equally

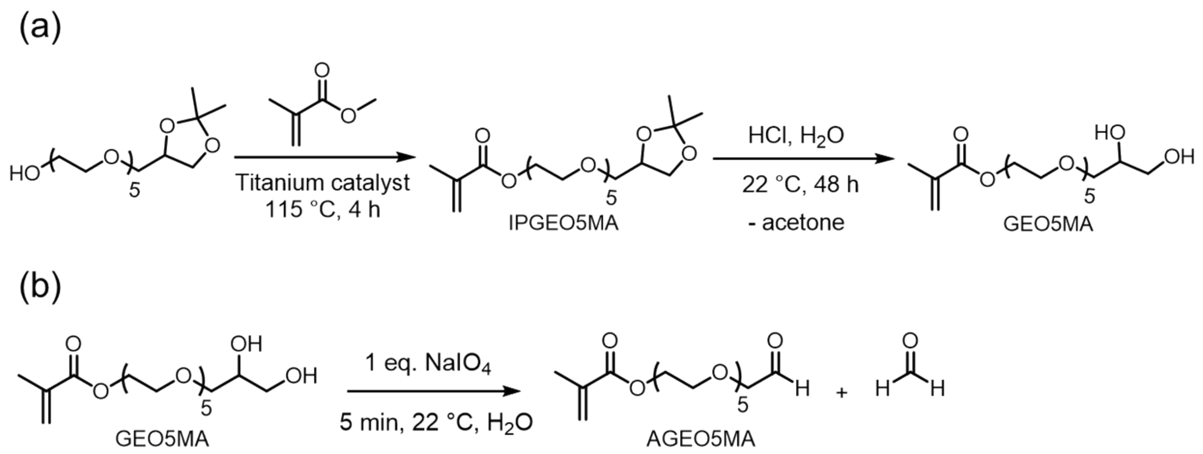

Scheme S1. (a) Two-step synthesis of GEO5MA monomer. The hydroxyl-capped oligo(ethylene glycol) isopropylideneglycerol precursor is transesterified with methyl methacrylate to produce IPGEO5MA, before removing the ketal protecting group with acid to afford GEO5MA monomer. (b) Selective oxidation of GEO5MA in aqueous solution using sodium periodate at 22 °C affords AGEO5MA with formaldehyde as a by-product. The same selective oxidation can be used to convert PGEO5MA homopolymer into PAGEO5MA homopolymer using identical reaction conditions.

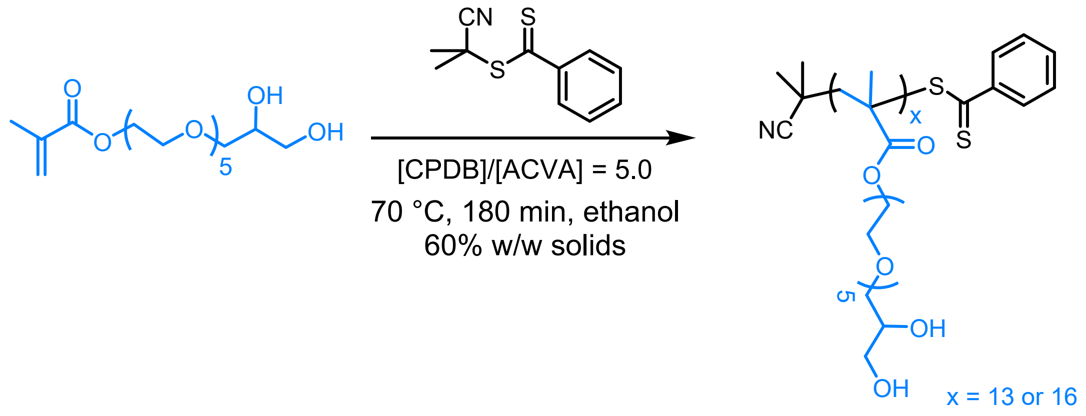

Scheme S2. Synthesis of PGEO5MA$_x$ homopolymer (x = 13 or 16) by RAFT solution polymerization in ethanol.

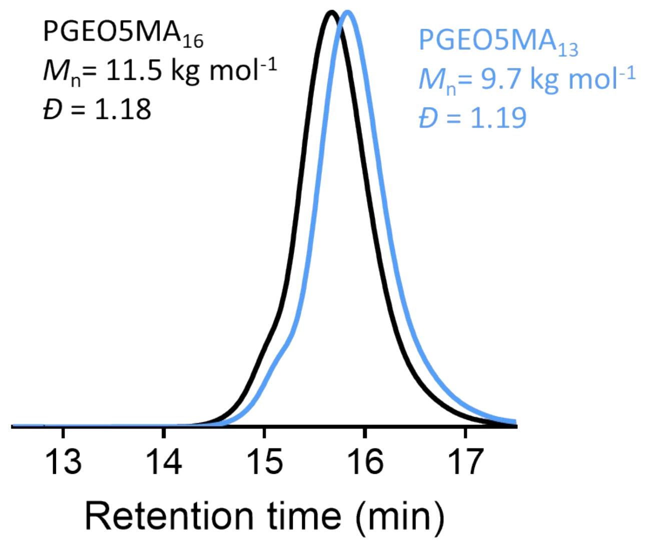

Figure S1. DMF GPC curves recorded for PGEO5MA$_{13}$ and PGEO5MA$_{16}$ (data expressed relative to a series of poly(methyl methacrylate) calibration standards).

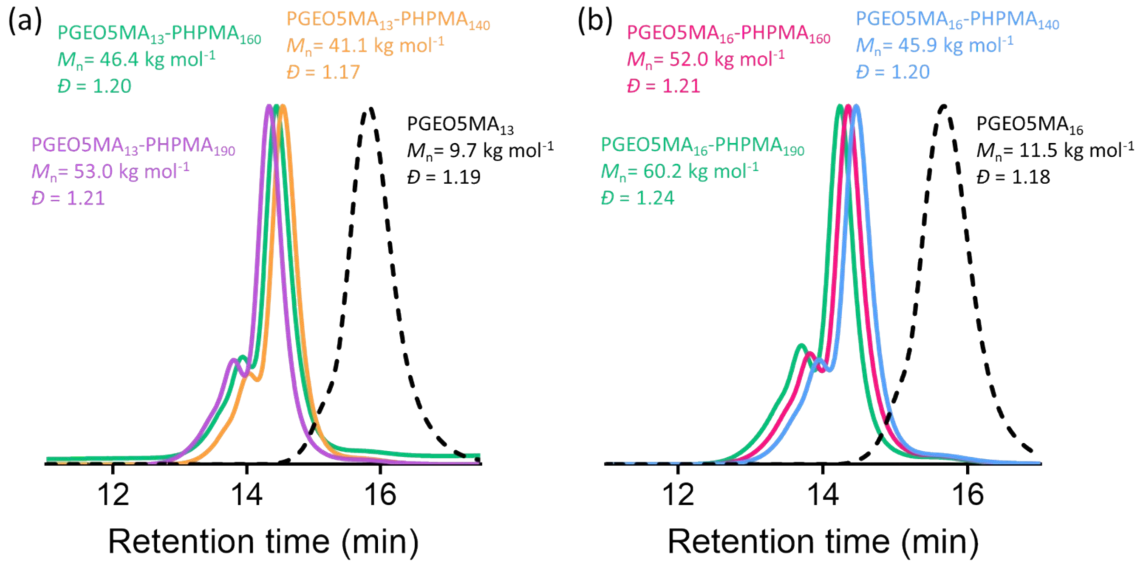

Figure S2. DMF GPC curves recorded for (a) PGEO5MA$_{13}$, PGEO5MA$_{13}$-PHPMA$_{140}$, PGEO5MA$_{13}$-PHPMA$_{160}$ and PGEO5MA$_{13}$-PHPMA$_{190}$ and (b) PGEO5MA$_{16}$, PGEO5MA$_{16}$-PHPMA$_{140}$, PGEO5MA$_{16}$-PHPMA$_{160}$ and PGEO5MA$_{16}$-PHPMA$_{190}$ (data expressed relative to a series of poly(methyl methacrylate) calibration standards).

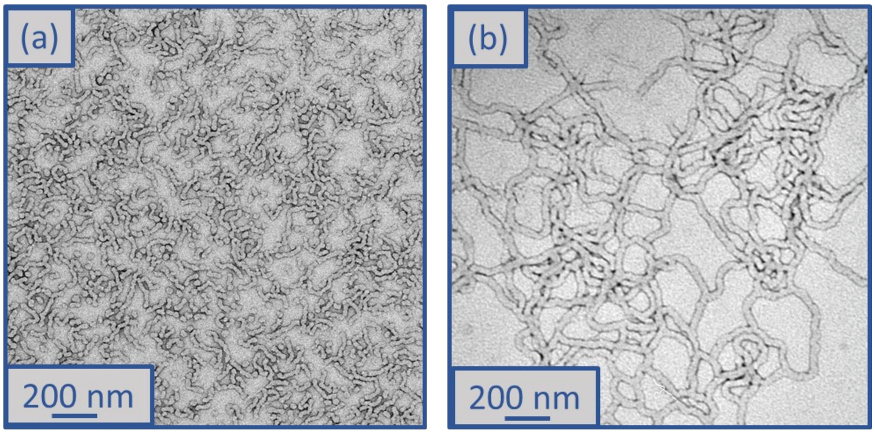

Figure S3. TEM images recorded for (a) PGEO5MA$_{13}$-PHPMA$_{150}$ and (b) PGEO5MA$_{13}$-PHPMA$_{190}$ worms.

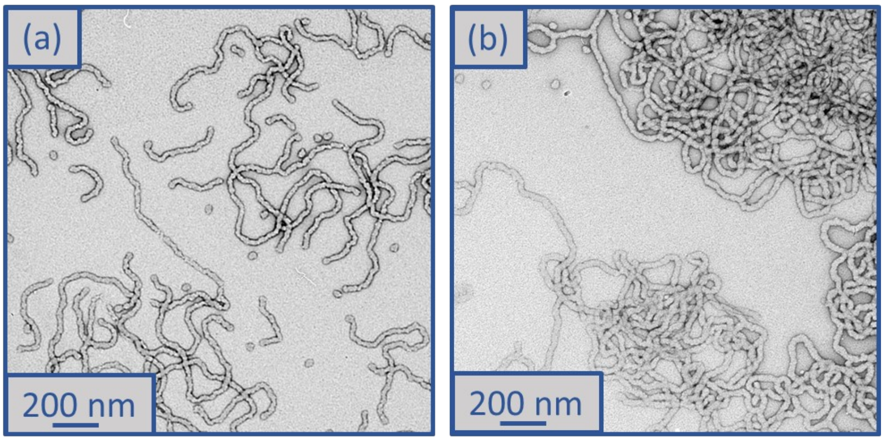

Figure S4. TEM images recorded for (a) PGEO5MA$_{16}$-PHPMA$_{170}$ and (b) PGEO5MA$_{16}$-PHPMA$_{200}$ worms.

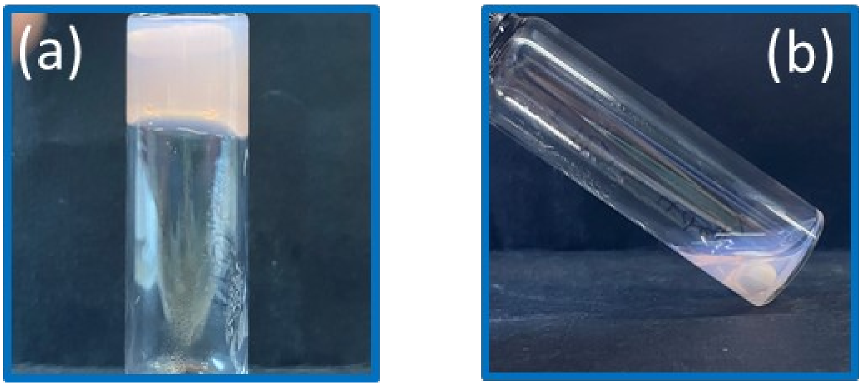

Figure S5. Digital photographs recorded for (a) a free-standing PGEO5MA$_{16}$-PHPMA$_{200}$ worm gel at 22 °C and (b) the same PGEO5MA$_{16}$-PHPMA$_{200}$ dispersion after cooling to 5 °C and then warming to 22 °C.

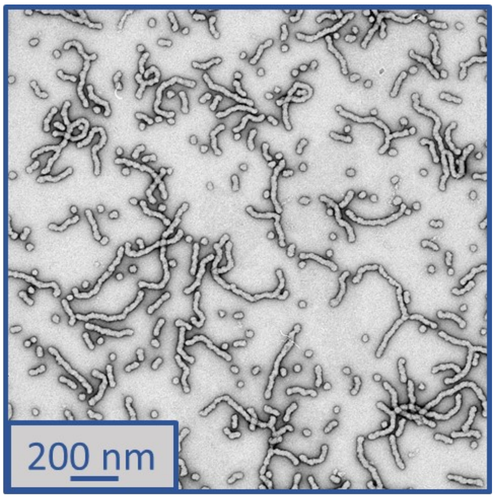

Figure S6. TEM image recorded for PGEO5MA$_{16}$-PHPMA$_{200}$ after a 22 °C to 5 °C to 22 °C thermal cycle.

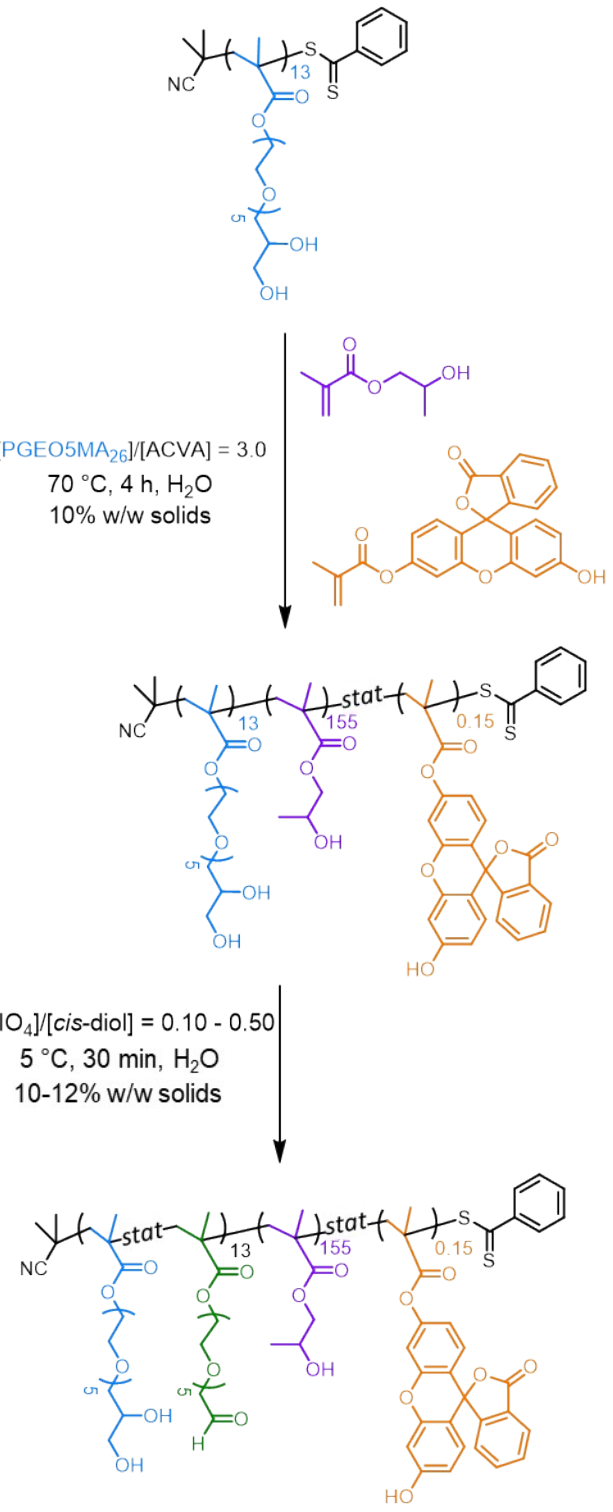

Scheme S3. Two-step synthesis of fluorescently-labeled aldehyde-functional PAGEO5MA$_{13}$-P(HPMA$_{155}$-*stat*-FMA$_{0.15}$) worms. First, a water-soluble PGEO5MA$_{13}$ precursor is chain-extended *via* RAFT aqueous dispersion statistical copolymerization of HPMA with 0.1 mol% FMA. In the second step, the pendent *cis*-diol groups on the PGEO5MA$_{13}$ stabilizer chains are selectively oxidized using a sub-stoichiometric amount of sodium periodate at 5 °C (NaIO$_4$/*cis*-diol molar ratios = 0.10 to 0.50).

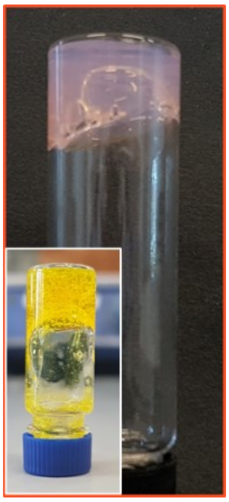

Figure S7. Digital photographs recorded for PGEO5MA$_{13}$-P(HPMA$_{155}$-*stat*-FMA$_{0.15}$) diblock copolymer worms, which form free-standing gels as judged by the tube inversion test. A pink worm gel was obtained at pH 5, whereas the inset indicates a bright yellow coloration for the same gel at pH 9 owing to the pH-sensitive fluorescent label (which is non-fluorescent at pH 5).

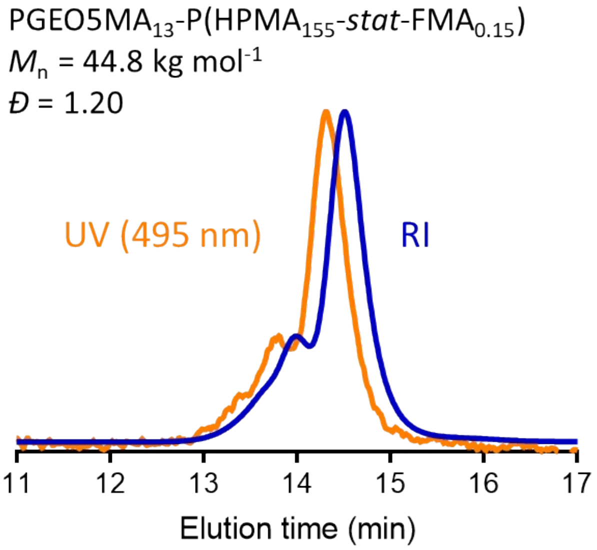

Figure S8. DMF GPC curves recorded using a refractive index (RI) detector (blue curve) and UV detector (wavelength set at 495 nm; orange curve) for PGEO5MA$_{13}$-P(HPMA$_{155}$-*stat*-FMA$_{0.15}$).

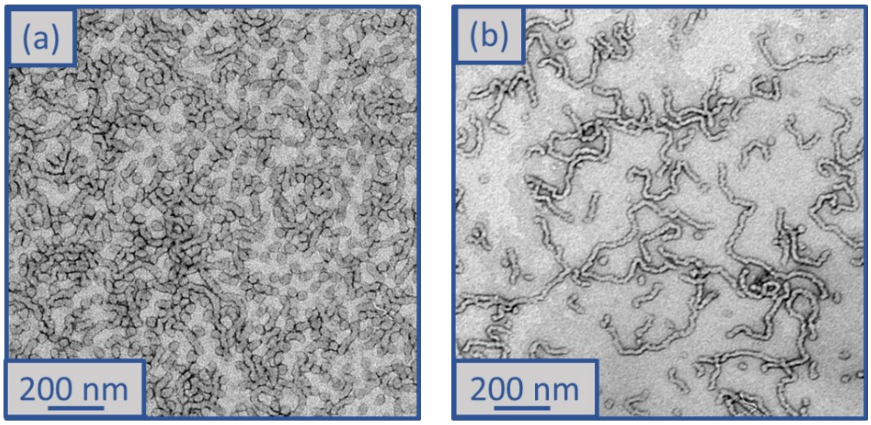

Figure S9. TEM images recorded for (a) PGEO5MA$_{13}$-PHPMA$_{155}$ and (b) PGEO5MA$_{13}$-P(HPMA$_{155}$-*stat*-FMA$_{0.15}$) diblock copolymer worms.

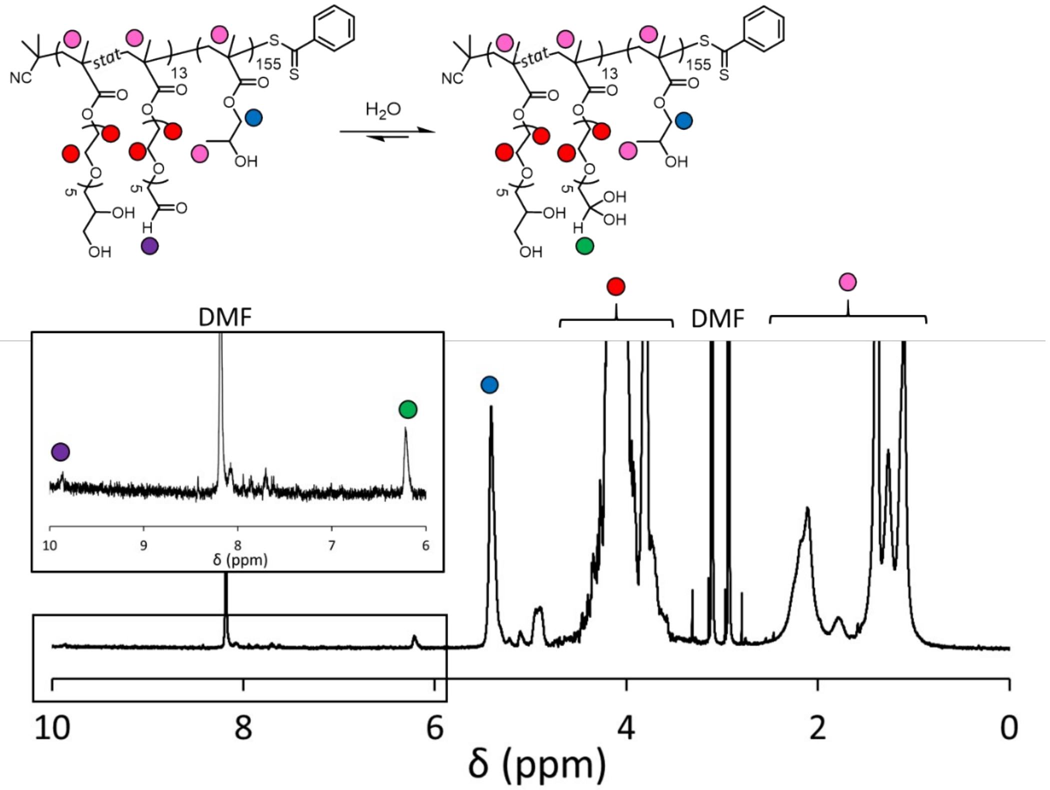

Figure S10. Assigned ^{1}H NMR spectrum (d_7-DMF) recorded for PGEO5MA$_{13}$-P(HPMA$_{155}$-*stat*-FMA$_{0.15}$) worms containing 30% aldehyde functionality.

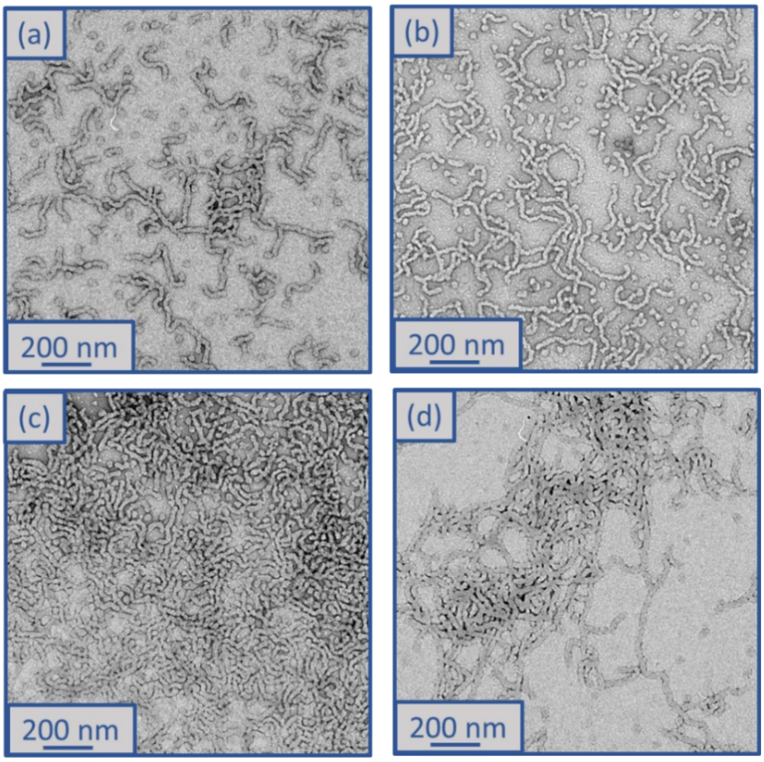

Figure S11. TEM images recorded for $PGEO5MA_{13}$-P($HPMA_{155}$-*stat*-$FMA_{0.15}$) worms containing (a) 10%, (b) 20%, (c) 30% or (d) 50% aldehyde functionality.

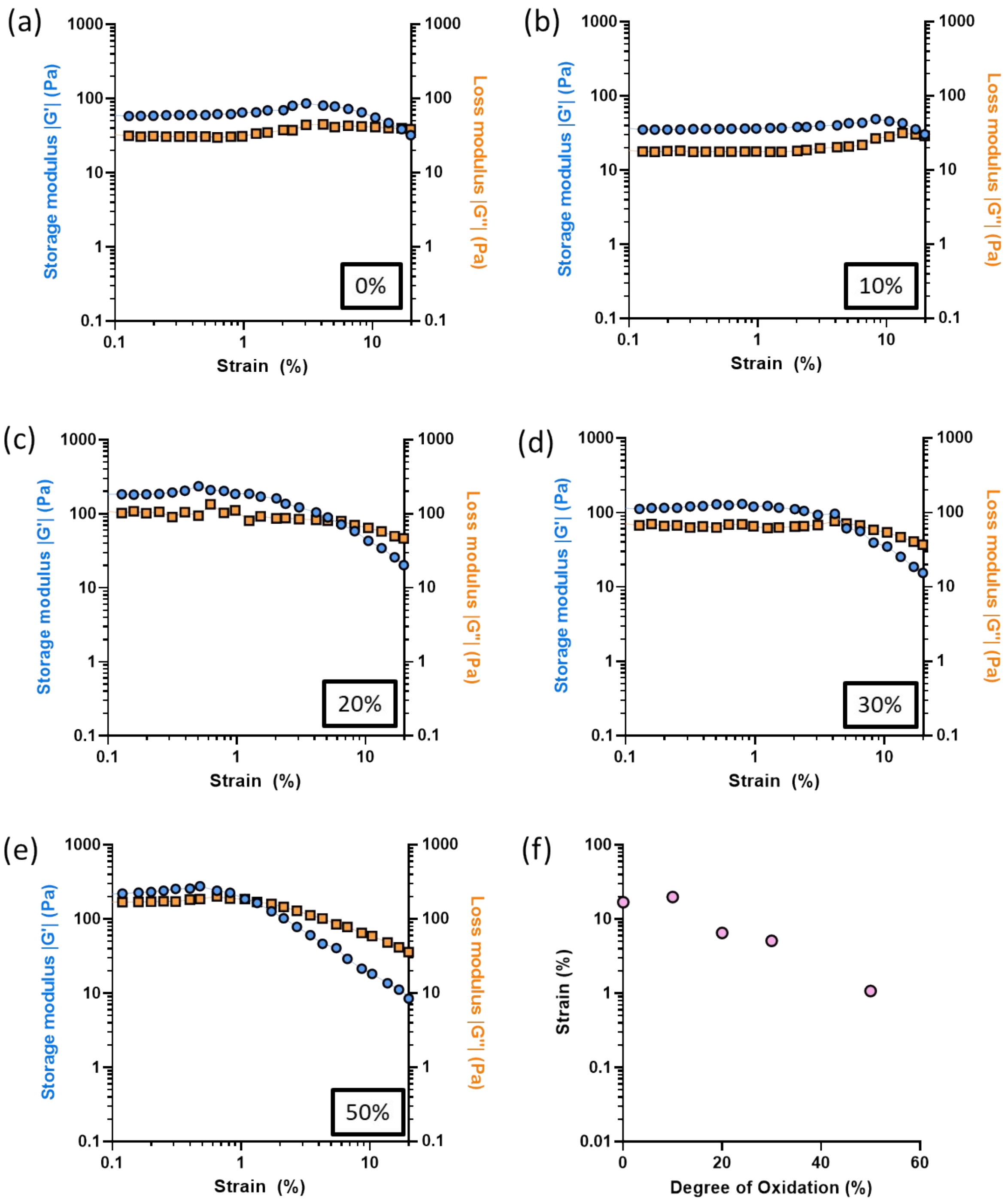

Figure S12. Storage and loss moduli (G' and G", respectively) vs. strain curves recorded at a constant angular frequency of 1.0 rad s^{-1} for $PGEO5MA_{13}$-P($HPMA_{155}$-*stat*-$FMA_{0.15}$) worms equilibrated at ambient temperature containing (a) 0%, (b) 10%, (c) 20%, (d) 30% and (e) 50% aldehyde functionality. (f) Plot of degelation strain% against the mean degree of oxidation (or aldehyde functionality).

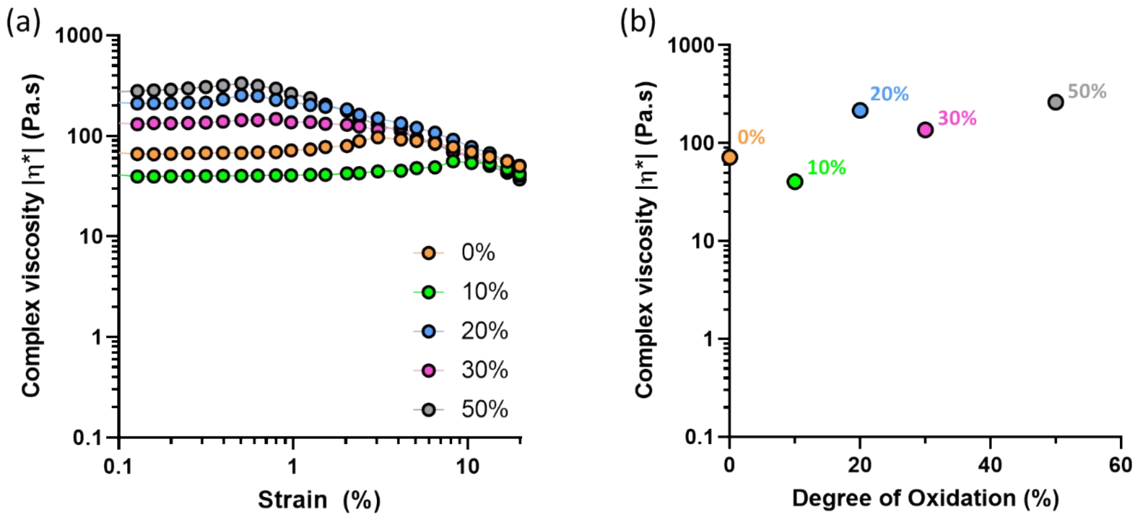

Figure S14. (a) Complex viscosity vs. strain curves recorded at a constant angular frequency of 1.0 rad s^{-1} for PGEO5MA$_{13}$-P(HPMA$_{155}$-*stat*-FMA$_{0.15}$) worms equilibrated at ambient temperature containing varying degrees of aldehyde functionality. (b) Plot of complex viscosity against mean degree of oxidation recorded at 1.0% strain.

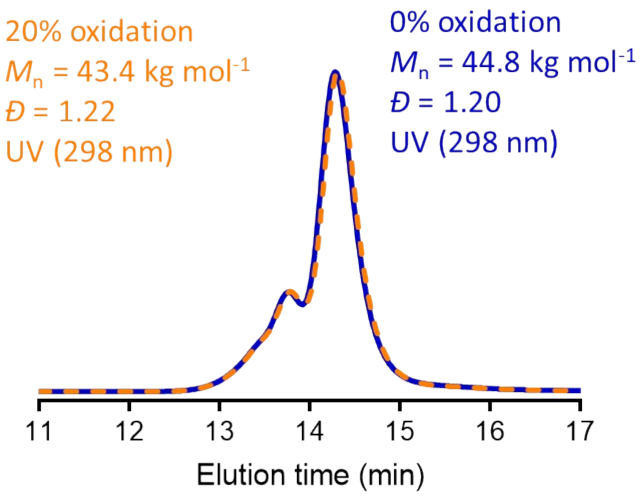

Figure S13. DMF UV GPC curves (UV detector wavelength set at 298 nm) for PGEO5MA$_{13}$-P(HPMA$_{155}$-*stat*-FMA$_{0.15}$) worms with either 0% or 20% aldehyde functionality.

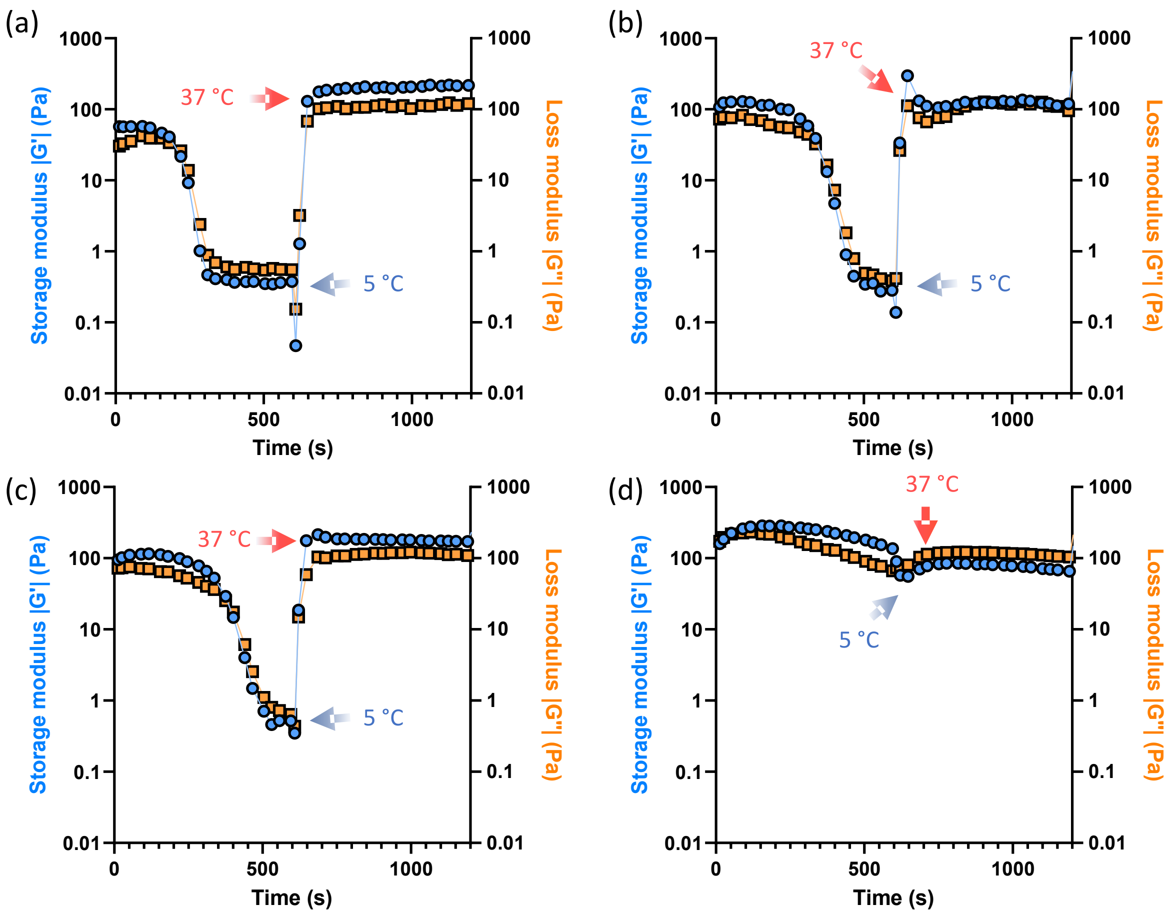

Figure S15. Time-dependent storage and loss moduli (G' and G", respectively) recorded *via* oscillatory rheology at either 5 °C or 37°C for PGEO5MA$_{13}$-P(HPMA$_{155}$-*stat*-FMA$_{0.15}$) worms containing (a) 10%, (b) 20%, (c) 30% and (d) 50% aldehyde functionality.

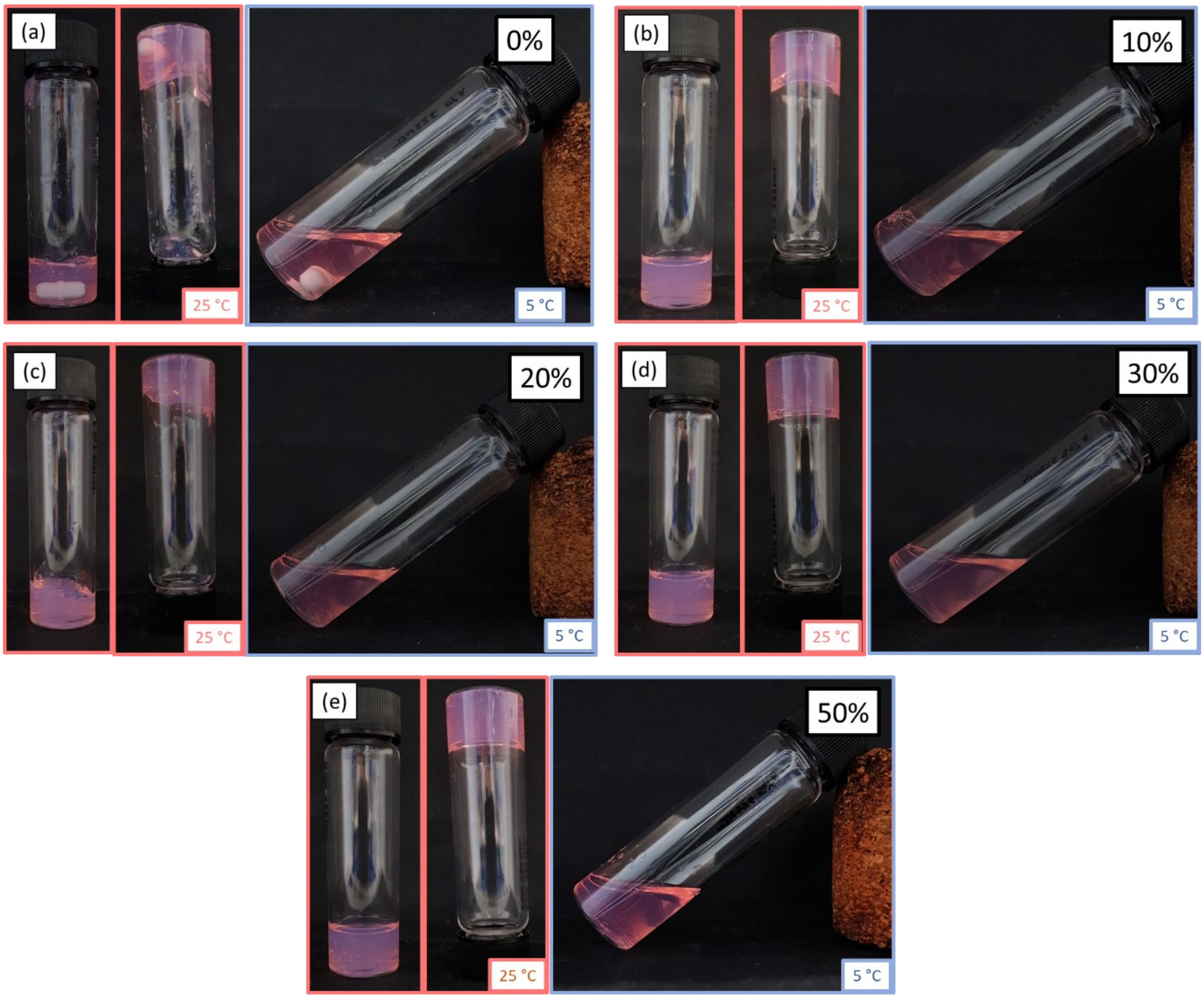

Figure S16. Digital photographs recorded for PGEO5MA$_{13}$-P(HPMA$_{155}$-*stat*-FMA$_{0.15}$) diblock copolymer nanoparticles at 25 °C and after cooling to 5 °C for (a) 0%, (b) 10%, (c) 20%, (d) 30% and (e) 50% aldehyde functionality.
